# Combining endangered plants and animals as surrogates to identify priority conservation areas in Yunnan, China

**DOI:** 10.1038/srep30753

**Published:** 2016-08-19

**Authors:** Feiling Yang, Jinming Hu, Ruidong Wu

**Affiliations:** 1 Institute of International Rivers and Eco-security, Yunnan University, Kunming, Yunnan 650091, China; 2 Yunnan Key Laboratory of International Rivers and Transboundary Ecosecurity, Yunnan University, Kunming, Yunnan 650091, China

## Abstract

Suitable surrogates are critical for identifying optimal priority conservation areas (PCAs) to protect regional biodiversity. This study explored the efficiency of using endangered plants and animals as surrogates for identifying PCAs at the county level in Yunnan, southwest China. We ran the Dobson algorithm under three surrogate scenarios at 75% and 100% conservation levels and identified four types of PCAs. Assessment of the protection efficiencies of the four types of PCAs showed that endangered plants had higher surrogacy values than endangered animals but that the two were not substitutable; coupled endangered plants and animals as surrogates yielded a higher surrogacy value than endangered plants or animals as surrogates; the plant-animal priority areas (PAPAs) was the optimal among the four types of PCAs for conserving both endangered plants and animals in Yunnan. PAPAs could well represent overall species diversity distribution patterns and overlap with critical biogeographical regions in Yunnan. Fourteen priority units in PAPAs should be urgently considered as optimizing Yunnan’s protected area system. The spatial pattern of PAPAs at the 100% conservation level could be conceptualized into three connected conservation belts, providing a valuable reference for optimizing the layout of the *in situ* protected area system in Yunnan.

To efficiently allocate limited resources to the most deserved or critical regions for biodiversity conservation, scientists have attempted to identify priority conservation areas (PCAs) using systematic conservation planning (SCP)^1^,^2^,^3^,^4^,^5^,^6^,^7^,^8^,^9^. Considering the complexity and limited knowledge of biodiversity across a variety of regions, as well as the time and cost required for data collection, the traditional method has been to select suitable surrogates to identify PCAs^1^,^7^,^10^,^11^,^12^,^13^,^14^,^15^,^16^,^17^,^18^,^19^,^20^,^21^. Previous studies^10^,^11^,^12^,^13^,^14^,^15^,^16^,^17^,^18^,^19^,^20^,^21^ have found that the effectiveness of surrogates in capturing the full range of biodiversity is partially dependent on a number of factors, including the target regions themselves, the types of surrogates used, the specific methodologies, and the scales of the units being studied. In addition, any one surrogate cannot perfectly represent all other biodiversity features. Previous studies have also shown that combining multiple taxonomic groups^9^,^10^,^12^,^13^,^16^ or physical environmental features (i.e., land use or ecosystem types)^11^,^13^,^14^,^16^,^17^,^18^,^19^,^21^ as joint surrogates can improve efficiency.

Yunnan encompasses several nationally or globally valued priority conservation areas^2^,^3^,^5^,^22^,^23^,^24^. Nearly half of China’s animal and plant species can be found in Yunnan^24^,^25^,^26^. According to the list of national key protected wild animals in China^27^ and the list of national key protected wild plants in China (first batch)^28^, Yunnan contains 192 key protected wild animals and 144 key protected wild plants, accounting for 59.5% and 47.8% of the total number of key protected wild animals and plants in China, respectively. In addition, the ancient origins of many Yunnan species indicate that the region has a high rate of endemism and a high ecological fragility^24^,^25^,^26^. Therefore, Yunnan plays an important role in conserving China’s biodiversity *in situ*.

Since the 1980s, Yunnan has established a complex *in situ* protected area system^29^,^30^,^31^. As of 2014, 159 nature reserves had been established, occupying approximately 7.4% of Yunnan’s land area^31^. However, studies have suggested that the current nature reserves in Yunnan are insufficient for capturing its total biodiversity^23^,^29^,^30^,^32^,^33^,^34^. The Yunnan Biodiversity Conservation Strategy and Action Plan (2012–2030)^33^ clearly revealed that Yunnan has critical conservation gaps, i.e., some biodiversity hotspots or priority areas are not protected adequately and some endangered national key protected species are not covered by any protected area. Recently, several studies have attempted to identify PCAs in Yunnan at both local^32^ and provincial scales^23^,^29^,^33^ using plant taxa. However, priority areas for animal conservation in Yunnan have not yet been investigated. Furthermore, the efficiency of the identified plant taxa-based PCAs in representing Yunnan animals was unclear. Therefore, identifying optimal PCAs for conserving both plants and animals in Yunnan and improving the current Yunnan protected area system (YPAS)^29^,^33^ are both extremely urgent.

County-level units (including counties, county-level cities and districts, hereafter all referred to as counties) in China are the main administrative bodies responsible for biodiversity conservation management. Statistics on the endangered plants and endangered animals (especially the latter) in counties in Yunnan are the only readily available and comparable species distribution data. Hence, this study selected non-uniform counties in Yunnan as the primary priority unit and used endangered plants and animals as surrogates to identify optimal PCAs for protecting endangered species in Yunnan in a cost-effective manner. We set three surrogate scenarios (endangered plants, endangered animals, and both endangered plants and animals) and two conservation levels (75% and 100% of the total number of endangered plants or/and animals in Yunnan). Through exploring the degree to which each type of PCAs was able to represent endangered plants or/and animals, we analysed the effectiveness of each surrogate scenario and determined the optimal scenario and its corresponding PCAs. We discussed the representativeness and conservation gaps of the optimal PCAs and the resulting implications for optimizing the current YPAS.

## Results

### Effectiveness of endangered plants or/and animals as surrogates

Plant priority areas (PPAs) included 2–3 more priority units than animal priority areas (APAs) at both the 75% and 100% conservation levels (Table 1). Correspondingly, PPAs covered approximately 2–3% more of Yunnan’s land area than APAs. The proportion of endangered animals protected by PPAs (incidental protection proportion) was 72.9% at the 75% conservation level and 87.5% at the 100% conservation level, while the proportion of endangered plants protected by APAs was only 41.7% and 77.6% at the two conservation levels, respectively. At the 75% conservation level, the proportion of overall endangered species (both plants and animals) conserved by PPAs was 12.5% higher than the proportion conserved by APAs. At the 100% conservation level, PPAs still conserved 2.7% more endangered species than APAs.

At the 75% conservation level, plant-animal priority areas (PAPAs) had only one more priority unit and accounted for an increase of only 1.0% of Yunnan’s land area compared to PPAs, but PAPAs conserved 19 more endangered plants and animals than PPAs; PAPAs required 3.07% more of Yunnan’s land area but conserved 61 more endangered plants and animals than APAs (Tables 1 and 2). At the 100% conservation level, the number and the total area of priority units in PAPAs were much higher than those in PPAs and APAs, but PAPAs also conserved 24 and 33 more endangered species than PPAs and APAs, respectively (Tables 1 and 2).

For better understanding the efficiency of coupled endangered plants and animals as surrogates, we merged the priority units of PPAs and APAs together at each conservation level to form a set that we designated merged priority areas (MPAs). At the 75% conservation level, both PAPAs and MPAs had eight priority units and accounted for a similar percentage of Yunnan’s land area. PAPAs, however, had a much higher efficiency (8.3%) in conserving endangered plants and a lower efficiency (1.0%) in conserving endangered animals than MPAs. Overall, PAPAs were 3.0% more efficient in conserving endangered species than MPAs. At the 100% conservation level, PAPAs had 5 fewer priority units and covered 7.3% less of Yunnan’s land than MPAs.

### Patterns of PPAs and APAs

**Figure 1:**
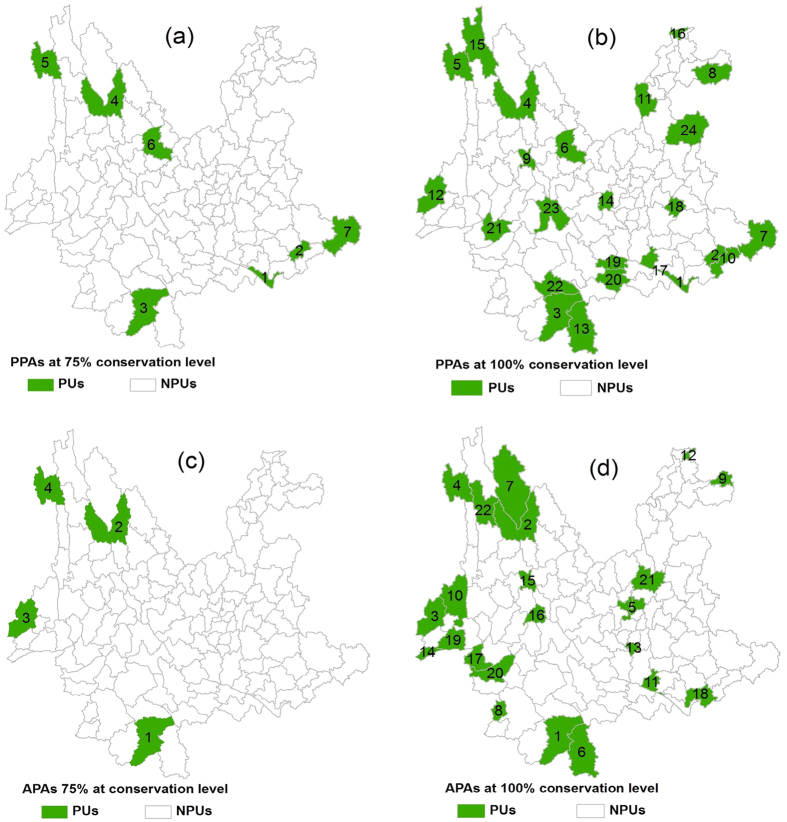
Distributions of PPAs and APAs at the 75% and 100% conservation levels. Priority units (PUs) were in green, while non-priority units (NPUs) were in white. The number in each priority unit represented its priority order: the smaller the number, the higher the priority. The distributions of PPAs were cited and modified from Yang *et al.*^23^. The map was created using ESRI ArcGIS 10.0 (http://www.esri.com/).

The patterns of PPAs at the two conservation levels followed Yang *et al.*^23^. For APAs, at the 75% conservation level, there were four dispersed units—two in the northwest, one in the south and one in southwest (Fig. 1c); at the 100% conservation level, APAs formed three clusters in the south, southwest, and northwest (Fig. 1d). A comparison of the PPAs and APAs at the 100% conservation level revealed that the northwestern and southern border regions were critical for both PPAs and APAs, while the southeastern border region was critical for PPAs (Fig. 1b) and the southwestern border region was critical for APAs (Fig. 1d).

The number of overlapping priority units and Jaccard similarity indexes at the two conservation levels (Table 1) indicated low spatial congruence between PPAs and APAs. At the 75% conservation level, PPAs covered three (numbered 1, 2 and 4 in Fig. 1c) of the four priority units in APAs, whereas APAs did not cover the former two critical priority units (numbered 1 and 2 in Fig. 1a) in PPAs. At the 100% conservation level, PPAs covered all of the former four critical priority units (numbered 1 to 4 in Fig. 1d) in APAs, but APAs still did not contain the former two critical priority units (numbered 1 and 2 in Fig. 1b) in PPAs.

### Patterns of PAPAs and MPAs

**Figure 2:**
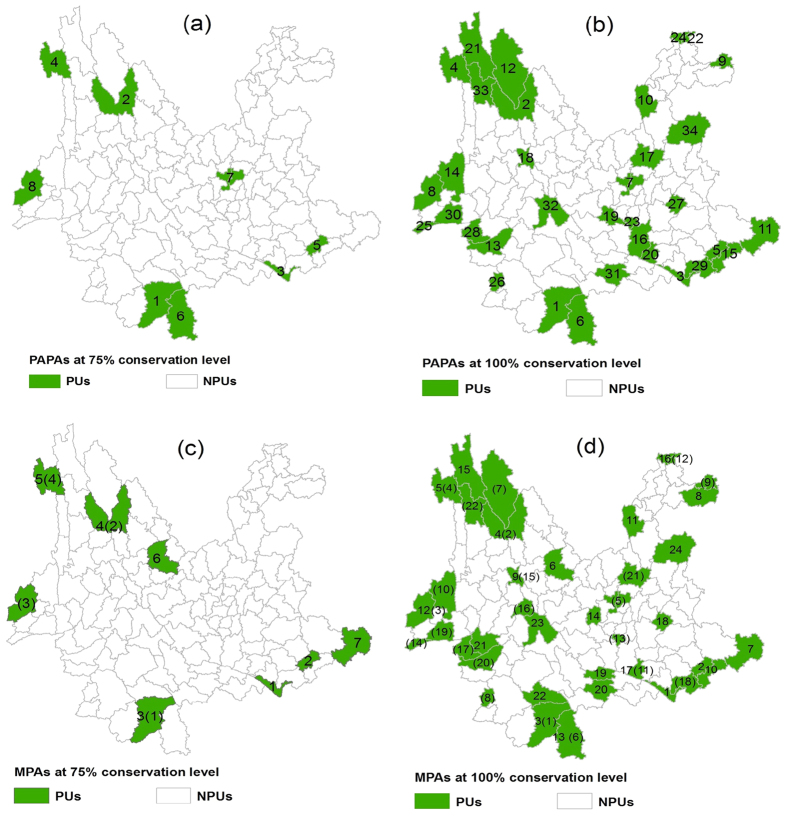
Distributions of PAPAs and MPAs at the 75% and 100% conservation levels. The number in each priority unit in (**a,b**) indicated its priority order: the smaller the number, the higher the priority. Because MPAs were merged by PPAs and APAs, the number before and within the parentheses in (**c,d**) represented the original priority orders of the units in PPAs and APAs, respectively, e.g., a unit labelled “3(1)” in (**c,d**) indicated that unit had a priority order of 3 in PPAs (Fig. 1a or 1b) and a priority order of 1 in APAs (Fig. 1c or 1d); a unit labelled “6” only in (**c,d**) indicated that unit had a priority order of 6 in PPAs (Fig. 1a or 1b) and was not the priority unit in APAs; a unit labelled “(8)” only in (**d**) indicated that unit was not the priority unit in PPAs and had a priority order of 8 in APAs (Fig. 1d). The map was created using ESRI ArcGIS 10.0 (http://www.esri.com/).

At the 75% conservation level, PAPAs included eight priority units, of which two were connected (numbered 1 and 6) and located in the south, while the other six were distributed sparsely in the southeast, northwest, southwest and central regions (Fig. 2a). At the 100% conservation level, PAPAs comprised five clusters: the southeast, south, northwest, southwest, and a south-central region (including units numbered 19, 23, 16, and 20, Fig. 2b). For MPAs, at the 75% conservation level, eight units were located in the northwest, southwest, southeast and mid-north (Fig. 2c), while at the 100% conservation level, four clusters were located in the south, southwest, northwest and southeast (Fig. 2d).

Overlapping priority units and the Jaccard similarity indexes at the two conservation levels (Table 2) indicated a higher spatial congruence between PAPAs and MPAs than between PPAs and APAs. Overall, PAPAs and MPAs appeared to include four similar clusters (southeast, south, northwest, and southwest) distributed along the national border belt as well as other priority units sparsely distributed along the central belt extending from northeastern to southwestern Yunnan. The major difference between the two was that PAPAs included one additional south-central cluster (including units numbered 19, 23, 16, and 20) extending southeastward from the central Yunnan plateau (Fig. 2b).

In addition, PAPAs at the 75% conservation level (Fig. 2a) covered all four critical priority units in APAs (Fig. 1c) and five of the seven critical priority units in PPAs (numbered 1 to 5, Fig. 1a). At the 100% conservation level, PAPAs (Fig. 2b) covered 21 of all 22 priority units (only excluding unit numbered 16, Fig. 1d) in APAs and 17 of all 24 priority units (excluding units numbered 6, 8, 14, 19, 21, 22, Fig. 1b) in PPAs. These results show that PAPAs at the two conservation levels covered the most critical priority units for conserving both endangered plants and animals in Yunnan.

## Discussion

### Does coupled surrogates improve effectiveness?

This study utilized endangered plants and animals as surrogates to identify PCAs at county level in Yunnan, China. As some previous studies have concluded^1^,^7^, our results revealed that endangered plants have a higher surrogacy value than endangered animals. However, the incidental protection proportion (Table 1) and spatial incongruence between PPAs and APAs (Fig. 1) clearly demonstrated that the two scenarios of surrogates cannot substitute for one another, but rather, complement each other. This complementarity may be caused to a certain extent by the correlation in spatial distribution between endangered plants and animals (cross-taxon correlation)^1^,^7^,^10^,^11^,^12^,^13^. Therefore, we were unable to use endangered plants or endangered animals alone as surrogate to identify a type of the PCAs that could fully satisfy the demand for overall biodiversity conservation in Yunnan.

Table 2 showed that at the 75% conservation level, PAPAs encompassed nearly the same land area as MPAs but conserved a greater number of endangered species. At the 100% conservation level, however, PAPAs required less land area than MPAs (Table 2). In addition, at both conservation levels, PAPAs covered the most critical priority units in both PPAs and APAs (Figs 1 and 2). These findings suggested that combining taxonomic groups into coupled surrogates could result in improved effectiveness compared to using a single taxonomic group as a surrogate^10^,^12^,^13^,^16^.

### Representativeness of PAPAs for overall species diversity patterns in Yunnan

The most important factor for the identified PCAs is how they represent regional species richness and endemism in target regions^5^. In Yunnan, there are three main plant species diversity centres, located in the southeast, south, and northwest^29^,^35^. Southeastern Yunnan is the centre of palaeo-endemic plants, while northwestern Yunnan is the centre of neo-endemic plants^35^,^36^,^37^,^38^. The richness of Yunnan vertebrates decreases generally from the national border belt to the central and northern part of Yunnan^34^. Along the national border belt, northwestern Yunnan is a major region of endemic (to China and/or Yunnan) vertebrates, southern and southeastern Yunnan contain endangered vertebrates, and southwestern Yunnan is home to both endemic (to China and/or Yunnan) and endangered vertebrates. For PAPAs at the 100% conservation level, four (southeast, south, southwest and northwest) of the five critical clusters showed high spatial congruence with the known centres of rich plant or animal diversity^29^,^35^, and these four centres were mainly located along the national border belt. These findings suggested that PAPAs identified based on the coupled endangered plants and animals to a large extent represented Yunnan’s overall species diversity distribution patterns.

### Representativeness of PAPAs on Yunnan’s biogeographic regions

**Figure 3:**
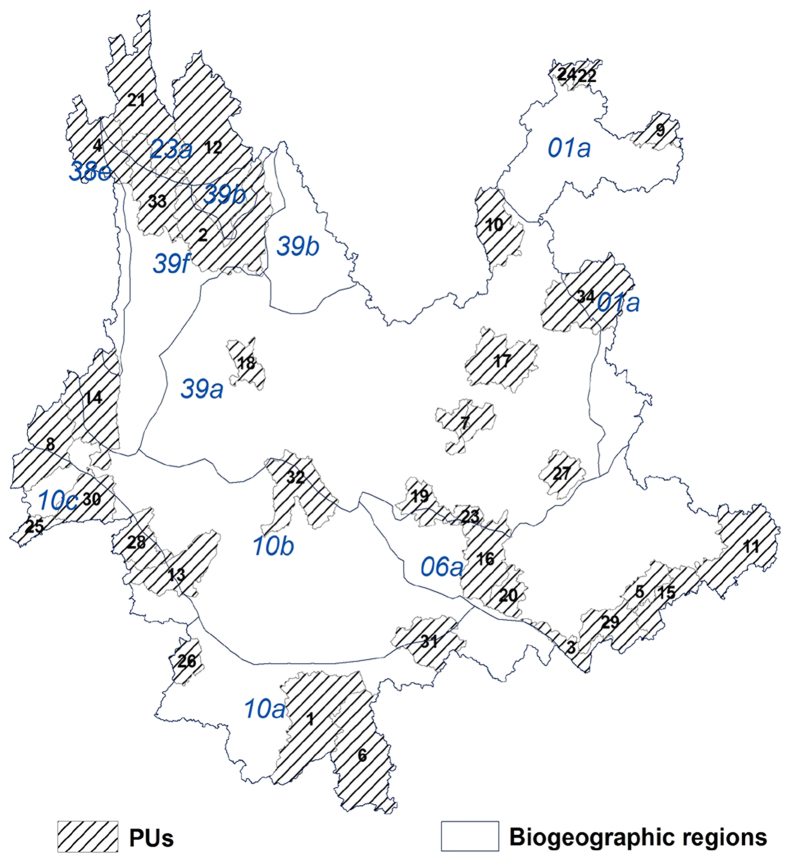
Spatial relationships between biogeographic regions (BRs) and PAPAs at the 100% conservation level. The number in each priority unit represented its priority order: the smaller the number, the higher the priority. The biogeographic regions were cited from and numbered according to Yang *et al.*^36^. The map was created with ESRI ArcGIS 10.0 (http://www.esri.com/).

According to Yang *et al.*^36^, Yunnan is the transition area between the Palearctic Realm (central to northern and southeastern Yunnan) and the Oriental Realm (central to southern and southwestern Yunnan), with the division line (in fact a transition or mixing belt) roughly running eastward from the middle of the Yunnan western border to central Yunnan and then turning southeastward along the mainstream Yuan River to the border. Under these two realms, Yunnan was divided into ten biogeographic regions (BRs)^35^. Figure 3 showed that the eight priority units in PAPAs at the 75% conservation level were scattered among seven BRs (BRs 10a, 39b, 06a, 38e, 39a, 10b and 10c), among which five (BRs 10a, 39b, 06a, 38e and 10c) were on the critical border belt. The figure also showed that at least one priority unit in PAPAs at the 100% conservation level fell completely or partially within each of the ten Yunnan BRs. For the critical border belt, northwestern Yunnan (covered by BRs 23a, 38e, 39b and 39f together), southwestern Yunnan (BRs 10c), southern Yunnan (BRs 10a and 10b), and southeastern Yunnan (southern part of BRs 06a) had five, five, four and nine complete or partial priority units, respectively. Therefore, PAPAs overlapped to a large extent with Yunnan’s most critical BRs.

### Implication of PAPAs for YPAS optimization

One of the critical stages and aims of SCP is to analyse coverage gaps and then optimize existing reserves network in the target region by using the identified PCAs^39^. As previous studies^1^,^40^ have noted, the priority units in PCAs identified by the Dobson algorithm with endangered species as surrogates at the county level can be taken as the approximate indication of priority units with geographically concentrated endangered species. Importantly, the Dobson algorithm characterizes their relative priority ordination. Hence, analysis of the current status of established protection areas of these priority units in PAPAs could reveal conservation gaps and help prioritize the optimization of these critical units for Yunnan biodiversity conservation. Table 3 showed that four priority units (numbered 7, 9, 27, 30) were not covered by any reserve, and ten priority units (numbered 2, 5, 11, 12, 16, 17, 19, 20, 26, 29) were covered by reserves with a coverage lower than 5%. These 14 priority units should be the focus of urgent attention in future YPAS optimization.

**Figure 4:**
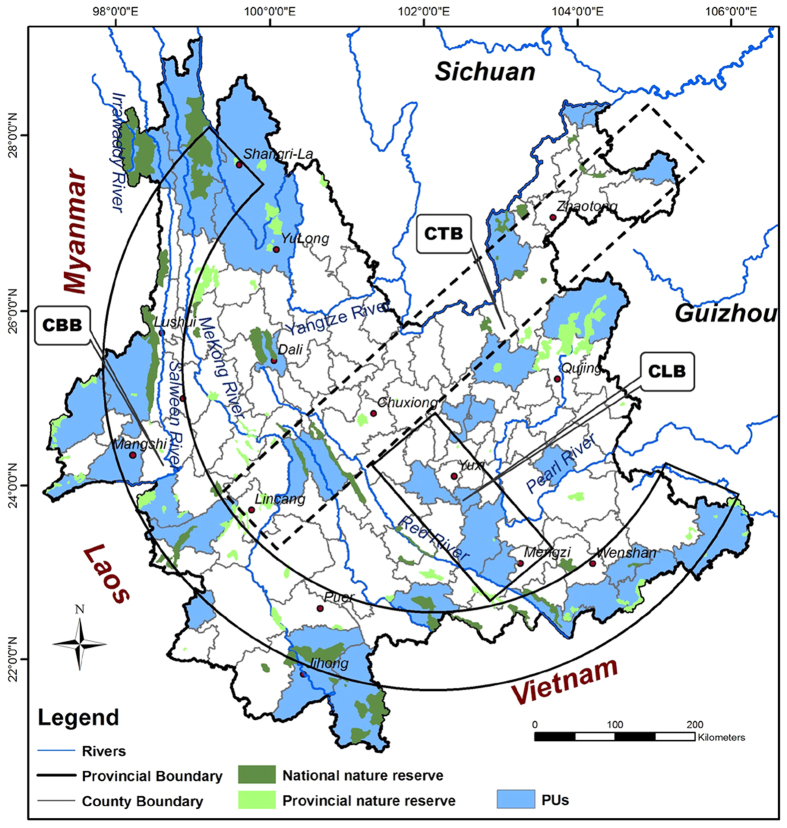
The macro spatial pattern of PAPAs at the 100% conservation level and existing nature reserves. The three connected belts were the C-shaped border belt (CBB) with four critical centres, the central transition belt (CTB) extending from northeastern Yunnan to southwestern Yunnan, and the central linking belt (CLB) forming a corridor between the CTB and the southeastern part of the CBB. The map was created using ESRI ArcGIS 10.0 (http://www.esri.com/).

Chen *et al.*^40^ found that the hotspots identified in county units (non-traditional ecological meaningful hotspots) had certain links with normally designated “hotspots” by Myers *et al.*^2^. In our study, we also found that PAPAs at the 100% conservation level well represented Yunnan’s overall species diversity distribution and critical biogeographical regions, which indicated that PAPAs had clear ecological meaning for biodiversity conservation in Yunnan. We conceptualized the macro spatial pattern of PAPAs at the 100% conservation level into three connected “belts” (Fig. 4). The first was a C-shaped border belt (CBB) with four critical centres. The second was a central transition belt (CTB), extending from northeastern Yunnan to southwestern Yunnan, which was partially spatially congruent with the central Yunnan biogeographic transition region^29^,^36^. The third was a central linking belt (CLB), forming a corridor between the CTB and the southeastern part of the CBB. The CBB has been highly valued by many studies for its roles in Yunnan biodiversity conservation^2^,^3^,^22^,^23^,^24^,^29^,^33^,^35^,^37^,^41^, while the values of CTB and CLB to Yunnan biodiversity conservation have been overlooked. These three connected belts held most critical priority units and established nature reserves (Fig. 4). Hence, we suggest that the macro spatial pattern of PAPAs at the 100% conservation level provides a valuable reference for priority units setting in Yunnan biodiversity conservation and the spatial layout optimization of the current YPAS. This was the first attempt to propose a macro pattern for YPAS optimization.

The methodology of this study followed the main principles of SCP^39^ with some differences. SCP emphasizes the contributions and commitments of existing reserves, first reviewing the status (especially coverage gaps) of existing conservation areas and then identifying additional conservation areas. This study emphasized the maximal conservation values and the complementarity of each unit to form a “notionally integral” conservation areas system for the target goal by first identifying and forming the optimal priority units set, then identifying the priority units with obvious coverage gaps (Table 3). Hence, the optimal PCAs (i.e., PAPAs) identified in this study provided a valuable reference pattern (three connected conservation belts) for optimizing Yunnan’s overall reserves network.

The county is not an ecologically meaningful space but an administrative unit. Considering environmental and species distribution heterogeneity, that a particular species occurs in a county does not indicate that a viable population can be maintained in that county^1^. However, the identified priority units can be taken as critical units with geographically concentrated endangered species in Yunnan. Through further coupling analysis with detailed environmental and ecological features, we can easily identify ecologically meaningful spaces in each priority unit of PAPAs. Fortunately, more detailed environmental and ecological features can now be easily derived from topographic, vegetation, or land use maps, along with remote sensing imagery and other tools. Combining counties with other environmental or ecologically meaningful units will provide more practical support for the optimization of regional conservation areas systems because county is the basic administrative body responsible for biodiversity conservation. Moreover, as traditional SCP suggests, incorporation of the opportunity costs (e.g., commercial uses from governmental planning) and pressures (e.g., current land use types and intensity, human population) into the identification of priority conservation units in a target region will provide a stronger basis for the optimization of regional conservation areas.

## Conclusions

Suitable surrogates are crucial for the identification of optimal PCAs in any target region. This study explored the efficiency of three surrogate scenarios in identifying PCAs at the county level in Yunnan, southwest China. Our analysis concluded that endangered plants had a higher surrogacy value than endangered animals but that the two were not substitutable. Compared to endangered plants or animals alone, coupled endangered plants and animals as surrogates improved the protection efficiency of the identified PCAs. Among the four types of PCAs, PAPAs had the highest protection efficiency and covered the most critical priority units for conserving both endangered plants and animals in Yunnan. Among these 34 priority units in PAPAs, four priority units were not covered by existing reserves and ten priority units had reserve coverage lower than 5%. PAPAs at the 100% conservation level were effective in representing overall species diversity (richness and endemism) distributions and critical biogeographical regions of Yunnan. The spatial pattern of PAPAs at the 100% conservation level was conceptualized into three connected belts (CBB, CTB and CLB), provided a reference for optimizing the macro pattern of *in situ* protected areas system in Yunnan.

## Methods

### Data

A total of 144 endangered plants and 192 endangered animals in Yunnan were included in our analysis^27^,^28^, and a spatial database of the distributions of these endangered species was compiled at the county level. Endangered plant species data sources were the same as those used by Yang *et al.*^23^. For endangered animals, the main source of information was the “China Species Information Service (CSIS)”^42^, with additional information from the “Yunnan Annals of Animals”^43^ and “The Animals Resource Database in the Southwest Region, China”^44^. Among these endangered species, 88 endangered plants and 35 endangered animals are endemic to China, 38 are first-order national protected plants and 43 are first-order national protected animals (Table 4), and 91.67% of endangered plants and 26.5% of endangered animals are IUCN Red List threatened species (CR, EN, VU) (Table 4). Nature reserve information (name, boundary, area, level, etc.) was compiled from the “Nature reserve annual report in Yunnan Province”^31^.

For geographical data, we derived the boundary of each county in Yunnan from the 1:250,000-scale Yunnan Geographic Information Database. Yunnan is currently composed of 129 counties. There have been some adjustments of administrative divisions in Yunnan since 2003. In 2003, Lijiang County was divided into Yulong County and Ancient Town District. In 2007, Cui’yun District (formerly Simao County) was renamed Simao District, and Pu’er County was renamed Ning’er County. In 2010, the city of Luxi became Mangshi. In 2011, Chenggong County was renamed Chenggong District of Kunming. The endangered species data for each county have been recorded since the 1980s, and the practice continues into the present; we used the administrative divisions that pre-date 2003 to maintain updated data. Kunming, therefore, was composed of four county-level districts (Wuhua, Panlong, Guandu and Xishan) in our database. In total, the boundaries of 125 counties were used in the analysis.

### Analysis

The key principle of the Dobson sorting algorithm is to identify optimal complementary subsets^1^. This study used the Dobson algorithm to identify priority conservation units at the county level in Yunnan. The algorithm first selected the county with the maximal number of surrogate species; all surrogate species found in that selected county were then excluded from further selection while the algorithm searched for the county with the maximal number of surrogate species not already selected^1^. This process was repeated until all selected counties met the specified conservation aim. In this process, if two or more counties had the same number of surrogate species, we selected the county with the smallest area. Hence, the Dobson algorithm can identify the optimal set of priority units that achieve the conservation aim with the smallest area. For the specified conservation goal in a planning region, the identified priority unit set is a sort of “notionally integral” conservation areas system.

We set three surrogate scenarios: endangered plants, endangered animals, and both endangered plants and animals. Based on these scenarios, we ran the Dobson algorithm and identify three types of PCAs directly. The three types of PCAs were referred to as plant priority areas (PPAs)^23^, animal priority areas (APAs), and plant-animal priority areas (PAPAs). We further merged all priority units of the PPAs and the APAs together to form a new set of priority units, which were termed merged priority areas (MPAs). We ran the Dobson algorithm at two target conservation levels (75% and 100%), meaning that each type of PCAs could conserve at least 75% and 100% of the total number of surrogate species, respectively, in Yunnan. Finally, we obtained four types of PCAs (PPAs, APAs, PAPAs and MPAs) at each conservation level.

To evaluate the efficiencies of the four types of PCAs, we calculated the percentage of Yunnan’s entire area covered by each type of PCAs and the number of surrogate species contained in each type of PCAs. The Jaccard similarity index^45^ was used to analyse the similarity of priority units between PPAs and APAs and between PAPAs and MPAs. Each of the four types was visualized on a map in ArcGIS 10.0 (ESRI Inc., 2010) to explore its spatial distribution pattern and overlapping priority units. All parameters were calculated and compared at both the 75% and 100% conservation levels.

## Additional Information

**How to cite this article**: Yang, F. *et al.* Combining endangered plants and animals as surrogates to identify priority conservation areas in Yunnan, China. *Sci. Rep.* **6**, 30753; doi: 10.1038/srep30753 (2016).

## Figures and Tables

**Table 1:** Protection efficiencies of the PPAs and the APAs.

Conservation levels	PCAs based on surrogate scenarios			Number (%^a^) of conserved endangered species			Number of overlapping priority units (similarity^b^)
	Types	Number of priority units	% of Yunnan land	Plants	Animals	Plants and Animals	
75%	PPAs	7	8.06	111 (77.08)	140 (72.92)	251 (74.70)	3 (0.38)
	APAs	4	6.02	60 (41.67)	149 (77.60)	209 (62.20)	
100%	PPAs	24	23.04	144 (100.00)	168 (87.50)	312 (92.86)	6 (0.15)
	APAs	22	20.67	111 (77.08)	192 (100.00)	303 (90.18)	

^a^Percentage relative to the total number of endangered plants, animals, and both.

^b^Jaccard similarity index.

**Table 2:** Protection efficiencies of the PAPAs and the MPAs.

Conservation levels	PCAs based on surrogate scenarios		Number (%^a^) of conserved endangered species				Number of overlapping priority units (similarity^b^)
	Types	Number of priority units	% of Yunnan land	Plants	Animals	Plants and Animals	
75%	PAPAs	8	9.09	125 (86.81)	145 (75.52)	270 (80.36)	6 (0.60)
	MPAs	8	9.08	113 (78.47)	147 (76.56)	260 (77.38)	
100%	PAPAs	34	31.25	144 (100.00)	192 (100.00)	336 (100.00)	32 (0.78)
	MPAs	39	38.58	144 (100.00)	192 (100.00)	336 (100.00)	

^a^Percentage relative to the total number of endangered plants, animals, and both.

^b^Jaccard similarity index.

**Table 3:** Status of existing nature reserves related to the priority units in PAPAs at the 100% conservation level in Yunnan.

Priority order	Priority Units (PUs) in PCAs		Existing Nature Reserves			
	Name	Area (km^2^)	Name	Level^a^	Area (km^2^)	% of PU area
1	Jinghong	7133	Xishuangbanna	National	1181.50	24.82
			Naban River	National	11.60	
			Jinghong	County	441.43	
			Bulong	Autonomous Prefecture	135.91	
2	Yulong (includes Ancient Town District)	7648	Lashi Lake	Provincial	65.23	4.25
			Yulong Snow Mountain	Provincial	260.00	
3	Hekou	1313	Dawei Mountain	National	311.68	23.87
			Nanxi River	Autonomous Prefecture	1.75	
4	Gongshan	4506	Gaoligong Mountain	National	1835.00	40.72
5	Xichou	1545	Wenshan	National	37.39	2.42
6	Mengla	7056	Xishuangbanna	National	1458.74	21.15
			Yiwu	Autonomous Prefecture	33.33	
7	Kunming	2190				
8	Yingjiang	4429	Tongbiguan	Provincial	224.79	5.07
9	Weixin	1416				
10	Qiaojia	3245	Yao Mountain	National	201.41	6.33
			Mashu	County	4.03	
11	Funing	5459	Tuoniang River	Provincial	191.28	3.50
12	Shangri-la	11613	Haba Snow Mountain	Provincial	219.08	3.31
			Bita Lake	Provincial	141.33	
			Napa Lake	Provincial	24.00	
13	Gengma	3837	Nangun River	National	264.21	10.87
			Lancang River	Provincial	152.81	
14	Tengchong	5845	Gaoligong Mountain	National	528.51	11.53
			Baihai Wetland	Provincial	16.29	
			Volcano geothermy park	County	129.90	
15	Malipo	2395	Lao Mountain	Provincial	205.00	9.84
			Laojun Mountain	Provincial	30.90	
16	Jianshui	3940	Swallow Cave	Provincial	16.01	0.41
17	Xudian	3966	Black-necked crane reserve	Provincial	72.17	1.82
18	Dali	1468	Cangshan Mountain and Erhai Lake	National	497.59	34.28
			Fengyang	Autonomous Prefecture	0.67	
			Butterfly Spring	Autonomous Prefecture	5.00	
19	Eshan	1972	Yubaiding	Autonomous Prefecture	69.62	3.53
20	Gejiu	1597	Dawei Mountain	National	13.78	0.96
			*Caryota urens* forest	County	1.60	
21	Deqin	7596	Baima Snow Mountain	National	2089.36	27.50
22	Shuifu	319	Tongluoba	Autonomous Prefecture	24.84	7.78
23	Tonghai	721	Xiu Mountain	County	92.69	12.86
24	Suijiang	882	Twenty-four Gang	Autonomous Prefecture	109.89	13.62
			Rare and endemic fish reserve in Suijiang of Jinsha River	County	10.24	
25	Ruili	1020	Tongbiguan	Provincial	108.18	10.61
26	Ximeng	1391	Fodian Mountain	County	13.50	3.99
			Mengsuo Dragon pool	County	42.00	
27	Luxi	1674				
28	Zhenkang	2642	Nanpeng River	Provincial	369.70	13.99
29	Maguan	2755	Gulinqing	Provincial	68.32	2.99
			Laojun Mountain	Provincial	14.19	
30	Mangshi	2987				
31	Lvchun	3167	Huanglian Mountain	National	618.60	19.53
32	Jingdong	4532	Ailaoshan Mountain	National	214.55	10.79
			Wuliang Mountain	National	274.62	
33	Weixi	4661	Baima Snow Mountain	National	727.04	15.60
34	Xuanwei	6257	Head Source of Pearl River	Provincial	1137.89	18.67
			Beipan River	Autonomous Prefecture	5.00	
			Aquatic animal reserve in Jinsha River	Autonomous Prefecture	25.00	

^a^Existing nature reserves are classified into four levels – national, provincial, autonomous prefecture, and county.

**Table 4:** The number of various protection criteria of national key protected wild species.

Endangered species	Groups	National protected criteria^a^		IUCN Red List criteria^b^							
		I	II	CR	EN	VU	LC	LC/NT	NT	DD	NE
Plants	Pteridophyte	3	23	17	7	2	0	0	0	0	0
	Gymnosperm	22	10	19	11	1	0	0	0	1	0
	Angiosperm	13	71	46	26	1	0	0	0	11	0
	Fungus	0	2	0	1	1	0	0	0	0	0
Animals	Mammals	23	24	2	8	19	0	6	2	3	7
	Herptiles	3	11	1	1	2	0	0	2	1	7
	Fish	1	3	1	0	0	0	0	0	0	3
	Insects	0	3	0	0	0	1	0	0	0	2
	Birds	16	108	6	1	10	89	0	8	0	10

^a^I - first-order national protected species in China; II - second-order national protected species in China.

^b^CR - critically endangered species in IUCN Red List criteria; EN - endangered; VU - vulnerable; LC - least concern; LC/NT - between least concern and near threatened; NT - near threatened; DD - data deficient; NE - not evaluated.
